# Deletion of transcription factor AP‐2α gene attenuates fibroblast differentiation into myofibroblast

**DOI:** 10.1111/jcmm.14421

**Published:** 2019-07-24

**Authors:** Gracious R. Ross, Stacie Edwards, Catherine Warner, Peter Homar, Francis X. Downey, Larisa Emelyanova, Farhan Rizvi, Arshad Jahangir

**Affiliations:** ^1^ Center for Integrative Research on Cardiovascular Aging Aurora Research Institute, Aurora Health Care Milwaukee Wisconsin; ^2^ Aurora Cardiovascular Services Aurora Sinai/Aurora St. Luke's Medical Centers Milwaukee Wisconsin

## 1 INTRODUCTION

Excessive fibrosis underlies many critical organ dysfunctions.^1^, ^2^ Fibrosis emanates from fibroblast trans‐differentiation into myofibroblasts,^3^ marked by increased α‐smooth muscle actin (α‐SMA) expression and excessive collagen secretion, initiated as a reparative process of normal wound healing and tissue repair in response to injury.^4^ However, activated myofibroblasts accumulate within pathological lesions of various fibrotic disorders,^5^ including patchy and interstitial fibrosis in progressive heart failure and cardiac hypertrophy.^6^ Therefore, attenuation of differentiation to myofibroblasts is expected to mitigate fibrosis. We attempted to find a potential target to extenuate the fibroblast differentiation by analysing the transcription factors in human fibroblasts/myofibroblasts, as transcriptome changes occur in fibroblasts during differentiation.^7^ Here, we report a novel molecular target, transcription factor AP‐2α (TFAP2A), to reduce fibroblasts trans‐differentiation.

## 2 MATERIALS AND METHODS

Informed consents were obtained from all participants, and the study was carried out according to the World Medical Association Declaration of Helsinki.

Human ventricular fibroblasts (hVFs)‐Control hVFs from disease‐free trauma victims (Lonza Inc, Allendale, NJ; ScienCell, Carlsbad, CA); hVFs were isolated from Heart Patients (Aurora Health Care, Milwaukee, WI), (HF) as reported earlier.^8^ NIH/3T3 fibroblasts (ATCC, Manassas, VA), Transforming growth factor (TGF)‐β1 (Peprotech, Rocky Hill, NJ), angiotensin II (Abcam, Cambridge, MA), miRNeasy Mini Kit, RT^2^ Profiler PCR Array, RT^2^ First Strand Kit, RT^2^ SYBR Green PCR master mix, miScript II RT kit (QIAGEN, Venlo, the Netherlands); Power SYBR Green PCR Master Mix (Thermo Fisher Scientific, Waltham, MA), Antibodies: Anti‐α‐SMA, Anti‐TFAP2A (Abcam, Cambridge, MA), Anti‐α/β‐tubulin and Anti‐GAPDH (Cell Signaling, Danvers, MA) were purchased.

### 2.1 Polymerase chain reaction array

**Figure 1:**
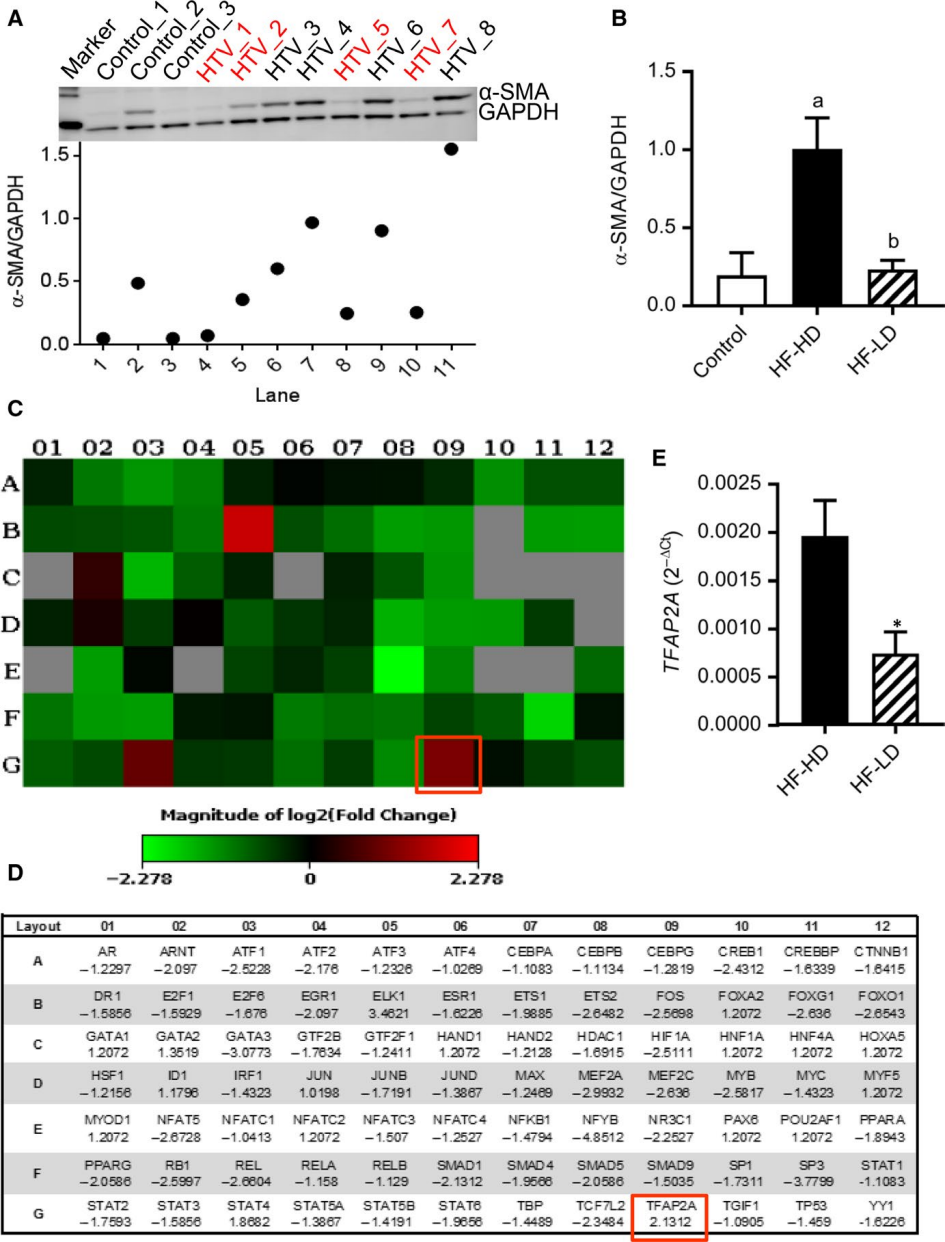
Expression of TFAP2A gene in human ventricular (myo)fibroblasts (hVFs) is significantly decreased with decrease in trans‐differentiation. Differentiation magnitude was assessed by the expression of α‐SMA, a marker of differentiated myofibroblasts. (A) Immunoblot displaying high and low expressions of α‐SMA in hVF lysates from each heart failure patients (HTV) [“red” label is patients with low differentiation], and control hVFs obtained from disease‐free trauma victims. Scattered plot displays the individual expression of α‐SMA normalized to GAPDH. (B) Bar graph displays analysis of grouped samples of high‐differentiation (HF‐HD, n = 4) and low‐differentiation (HF‐HD, n = 4), based on the α‐SMA/GAPDH ratio compared to the control (n = 3) group. (C) The Heat Map visualizing the fold changes in expression of genes in the Transcription factor qPCR Array between HF‐LD and HF‐HD group hVFs. Note that TFAP2A gene (well: G09) expression is significantly different between the two groups (n = 3). (D) Table provides the fold regulation (vs HF‐LD) data used for the map associated with each gene. (E) Validation of TFAP2A expression in hVFs from HF‐HD and HF‐LD patients by qRT‐PCR (2^‐ΔCt^). ^a^ *P *= 0.014 vs Control; ^b^ *P *= 0.01 vs HF‐HD; one way–ANOVA followed by Tukey's multiple comparisons test. *P < 0.05, n = 5; unpaired *t* test

The isolated hVFs were grouped into fibroblasts‐less differentiated (HF‐LD) and fibroblasts‐highly differentiated (HF‐HD) based on their α‐SMA expression (immunoblot), compared to the control hVFs (Figure 1A,B). Polymerase chain reaction (PCR) array was performed with *RT* ^2^ Profiler™ PCR Array‐Human Transcription Factors and compared between HF‐LD (n = 3) and HF‐HD (n = 3). Mature RNA (miRNeasy Mini kit) was reverse transcribed using RT^2^ First strand cDNA synthesis kit. The cDNA was used on the real‐time *RT* ^2^ Profiler PCR Array (QIAGEN, Cat# PAHS‐075Z) in combination with RT^2^ SYBR® Green qPCR Mastermix (Roche LightCycler® 480 Instrument). Threshold cycle (C_T_) values (excel file) were uploaded onto the data analysis centre web portal (http://www.qiagen.com/geneglobe). C_T_ values were normalized based on a Manual Selection of reference genes. The fold change/regulation (2^(‐ΔΔC_T_)) was calculated using ΔΔC_T_ method [ΔC_T_ was calculated between target gene and an average of reference genes (HKG), followed by ΔΔC_T_ calculations (ΔC_T_ (Test Group)‐ΔC_T_ (Control Group))].

### 2.2 Quantitative real‐time PCR

Total RNA was isolated from hVFs (miRNeasy Mini kit) and reverse transcribed (miScript RT II kit) with the supplied HiFlex buffer. qPCR was performed on the LightCycler 480 Instrument II, using the Power SYBR Green PCR Master Mix and 10 ng diluted cDNA per well. The following human primers were used: *TFAP2A*—F:5′‐GACCTCTCGATCCACTCCTTAC‐3′ R: 5′‐GAGACGGCATTGCTGTTGGACT‐3′; β‐2‐microglobulin (*B2M)*‐ F: 5′‐CCACTGAAAAAGATGAGTATGCCT‐3′ and R: 5′‐CCAATCCAAATGCGGCATCTTCA‐3′. The following PrimeTime qPCR mouse primer assays were used: *α‐SMA* (Mm.PT.58.16320644); *COL1A1* (Mm.PT.58.7562513); *COL2A1* (Mm.PT.58.5206680); *COL3A1* (Mm.PT.58.13848686), *TGFBR1* (Mm.PT.58.28402453), *TGFBR2* (Mm.PT.58.6358355) and *B2M* (Mm.PT.39a.22214835). The cycling conditions were 95°C for 10 minutes, followed by 40 cycles at 95°C for 15 s, 1 minute at 60°C, and 72°C for 40 s. Melt curve analysis was performed by an additional dissociation step of 1 cycle at 95°C for 5 s followed by 65°C for 1 min and ramping data collection to 97°C. Relative expression values (ΔCt) were obtained by normalizing Ct values (Roche Lightcycler 480 Software v1.5.1.62) of the tested genes with that of *B2M*.

### 2.3 CRISPR/CAS9‐based deletion of TFAP2A gene

The TFAP2A knockout cell line with NIH/3T3 fibroblasts (TFAP2A‐KO) was established using CRISPR/CAS9 technology through *Creative Biogene,* Shirley, NY.

### 2.4 In vitro trans‐differentiation protocol

Fibroblasts from wild‐type or TFAP2A‐KO groups were plated at 4000 cells/cm^2^ with DMEM media (10% BCS) and incubated at 37°C under 5%CO_2_. Following 24 hours, hVFs were either treated with TGF‐β1 (5 ng/mL), angiotensin II (100 nM) or kept as control in DMEM media (2.5% BCS). After 48‐72 hours, the fibroblasts/myofibroblasts were rinsed with Dulbecco's PBS and assayed.

### 2.5 Immunoblotting

Standard western protocols were followed^8^ with respective primary (dilutions: α‐SMA, 1:500, TFAP2A, 1:100) and secondary antibodies (1:2000). All samples were immunoblotted simultaneously and repeated at least twice.

### 2.6 Proliferation

Both WT and KO fibroblasts were plated as stated before in triplicate (per time‐point) in 6‐well plates and counted by Cellometer Auto 2000 (Nexcelom Bioscience, Lawrence, MA) at 24, 48, and 72 hours post‐plating. Doubling time was calculated by [*t *−* t*_0_]/{[log(N*_t_*)‐log(N_0_)]/log(2)}, where *t*_0_ refers time (initial count), *t* represents time (second count), N_0_ refers count at time t_0_, and N*_t_* represents count at time *t*.

## 3 RESULTS AND DISCUSSION

**Figure 2:**
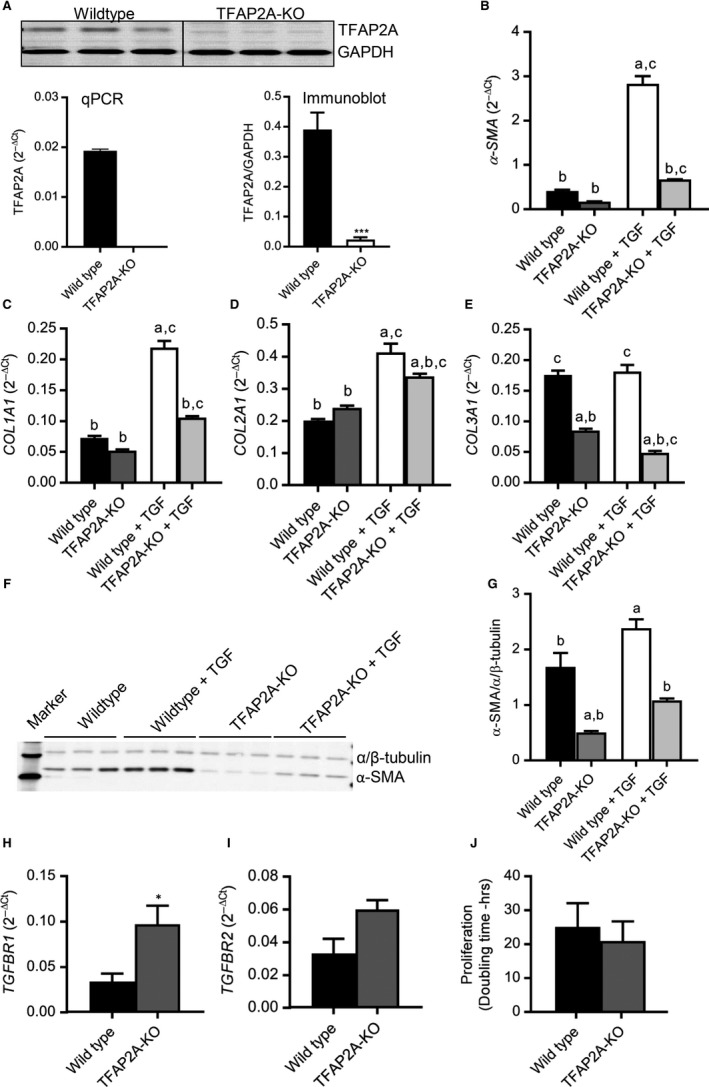
Deletion of TFAP2A gene significantly reduces TGF‐β1‐induced fibroblast differentiation. (A) CRISPR/Cas9‐based gene editing in NIH/3T3 fibroblasts deleted the TFAP2A expression, as validated by pooled Real‐time PCR data (2^‐ΔCt^) of *TFAP2A* gene (normalized to *B2M* gene) and immunoblotting between NIH‐3T3 (wild‐type) and TFAP2A‐knocked out (TFAP2A‐KO) fibroblasts. Gene expression of α‐smooth muscle actin (*α‐SMA*), collagen (COL) 1A1 (*COL1A1*), *COL2A1*, and *COL3A1 *were quantitatively analysed by real‐time PCR in wild‐type and TFAP2A‐KO fibroblasts. Incubation in TGF‐β1(5 ng/ml for 48‐72h) significantly increased the expression of *α‐SMA* (B), *COL1A1* (C), *COL2A1*(D) in the wild‐type with muted effect in the TFAP2A‐KO fibroblasts. While TGF‐β1 did not have any significant effect on the *COL3A1*(E) expression in the wild‐type, *COL3A1* expression was significantly down‐regulated both at basal level and following TGF‐β1 treatment in the TFAP2A‐KO fibroblasts. Immunoblot (F) and the bar graph(G) show that TGF‐β1 significantly increased the α‐SMA protein expression in the wild‐type with muted effect in the TFAP2A‐KO fibroblasts. TGF‐β1 receptor type1 (*TGFBR1*) mRNA levels were increased in TFAP2A‐KO (H) with no significant difference in type 2 (*TGFBR2*) (I) compared to wild‐type fibroblasts. (J) Proliferation rate (doubling time) was not significantly different between the groups. ^a^ *P* < 0.05 vs wild‐type, ^b^ *P* < 0.05 vs wild‐type + TGF, ^c^ *P* < 0.05 vs TFAP2A‐KO groups; n = 3, One‐way ANOVA followed by Tukey's multiple comparisons test. *P < 0.05, unpaired *t* test.

From left ventricle of human heart, fibroblasts were isolated and grouped into less differentiated (HF‐LD) and highly differentiated (HF‐HD) based on their α‐SMA expression, compared to control hVFs as shown in Figure 1A,B. PCR array of human transcription factors uncovered that the *TFAP2A* expression, along with ELK1, was decreased with decrease in differentiation as visualized in the heat map (Figure 1C) and fold regulation data (Figure 1D) (n = 3). This decreased expression of *TFAP2A* in HF‐LD fibroblasts compared to HF‐HD myofibroblasts noticed in PCR array was validated by quantitative reverse transcriptase‐PCR (n = 5) (Figure 1E). Based on these data, we have suggested that TFAP2A is crucial for the trans‐differentiation of fibroblasts into myofibroblasts. We applied CRISPR/Cas9‐based gene editing to knockout TFAP*2A* from NIH/3T3 fibroblasts (Figure 2A) and analysed the differentiation‐ and pro‐fibrotic parameters at both basal level and following TGF‐β1 treatment. TGF‐β1 significantly increased the mRNA expression of *α‐SMA* (Figure 2B), collagen (*COL*) *1A1* (Figure 2C), *COL2A1* (Figure 2D) in the wild‐type while the TGF‐β1 effect was significantly low in the TFAP2A‐KO fibroblasts. Even at basal level, the expressions of *α‐SMA* (Figure 2B) and *COL3A1* (Figure 2E) were significantly decreased in the TFAP2A‐KO fibroblasts compared to the wild‐type. This suggests that TFAP2A is important for the trans‐differentiation of fibroblasts to myofibroblasts. This reduced differentiation of TFAP2A‐KO fibroblasts observed in qPCR was further confirmed at protein level by immunoblotting where α‐SMA expression was significantly low both at basal level and after TGF‐β1 administration (Figure 2F,G). The blunted effect of TGF‐β1 in the TFAP2A‐KO fibroblasts does not appear to be due to changes in the upstream TGF‐β1 receptor levels, as the mRNA levels of TGF‐β1 receptor type1 (*TGFBR1*) is increased in TFAP2A‐KO fibroblasts (Figure 2H) without any significant difference in the type2 receptors (*TBFBR2*) compared to the wild‐type (Figure 2I). Interestingly, deletion of TFAP2A gene attenuates not only TGF‐β1‐induced fibroblast differentiation, but also angiotensin II (Ang II)‐induced differentiation as well, as evident from lack of increase in α‐SMA expression in the TFAP2A‐KO fibroblasts (Supplemental Figure). This suggests that TFAP2A could serve as a common downstream regulator of genes associated with fibroblast differentiation. Importantly, the knockdown of *TFAP2A* did not adversely affect the basal proliferation capacity (Figure 2J). The TFAP2A‐KO fibroblasts proliferated like that of the wild‐type with a doubling time of 21 ± 6 hr (TFAP2A‐KO) vs 25 ± 7 hr (wild‐type) (n = 3). TFAP2A is a known DNA‐binding transcription factor to have both repressive and facilitating effects^9^ on various genes and complete knockout of which is embryonically lethal.^10^ The exact mechanism for the reduced trans‐differentiation of TFAP2A‐KO fibroblasts in response to TGF‐β1 is unclear. Chromatin immunoprecipitation studies of Smad2/3, important factors in TGF‐β1 signalling, revealed abundant TFAP2A binding elements in Smad2/3 binding sites of the promoter regions of various genes in keratinocytes and knockdown of TFAP2A changed the TGF‐β1‐ induced transcriptions.^11^ Whether similar mechanisms underlie in fibroblasts is not known. In human Sertoli cells, Bone Morphogenetic Protein (BMP) 6, a member of TGF‐β superfamily, targets TFAP2A to positively regulate their growth.^12^ In contrast, the basal proliferation of fibroblasts did not reduce following *TFAP2A* knockdown in our study. This is in accordance with the observation in another study where TFAP2A can induce cell cycle arrest^13^ while reduced TFAP2A expression was suggested to impair p21cip‐mediated growth arrest resulting in increased proliferation.^14^ These properties found in the TFAP2A‐KO fibroblasts suggest that TFAP2A could emerge as a useful molecular target to mitigate excessive fibrosis by inhibiting fibroblast differentiation. As evident from the isolated human cardiac fibroblasts from left ventricles, the decrease in TFAP2A expression when cardiac fibroblast differentiation is decreased, suggest that TFAP2A is crucial for the trans‐differentiation of cardiac fibroblasts into myofibroblasts which can lead to excessive cardiac fibrosis underlying many cardiac dysfunctions. Therefore, selective inhibition of TFAP2A could develop as a novel therapeutic strategy to reduce cardiac fibroblast differentiation into myofibroblast, mitigate cardiac fibrosis and preserve cardiac function.

## CONFLICT OF INTEREST

There is no conflict of interest.

## AUTHORS’ CONTRIBUTIONS

GRR initiated, designed, executed, analysed the study and wrote the manuscript; SE executed the real‐time PCR and PCR array; CW and PH implemented the cell culture, immunoblotting and proliferation assays; FXD, LE, FR and AJ interpreted data and proof‐read the manuscript.

## Data Availability

All data sets are publicly available from the Dryad Digital Repository at https://doi.org/10.6084/m9.figshare.7898168.
