# Supplementary material for: Deletion of transcription factor AP‐2α gene attenuates fibroblast differentiation into myofibroblast
Source: J Cell Mol Med. 2019 Jul 24;23(9):6494–8. doi: 10.1111/jcmm.14421 (PMC6714505; doi:10.1111/jcmm.14421)

**Supplemental Fig:** Deletion of TFAP2A gene significantly reduces Angiotensin II –induced fibroblast differentiation. Immunoblots and the bar graph show that angiotensin II (100 nM for 72h) increased the α-SMA protein expression in the wild type with muted effect in the TFAP2A-KO fibroblasts. ^a^P<0.05 vs wildtype, ^b^P<0.05 vs wildtype + AngII, n=3, One-way ANOVA followed by Tukey’s multiple comparisons test.


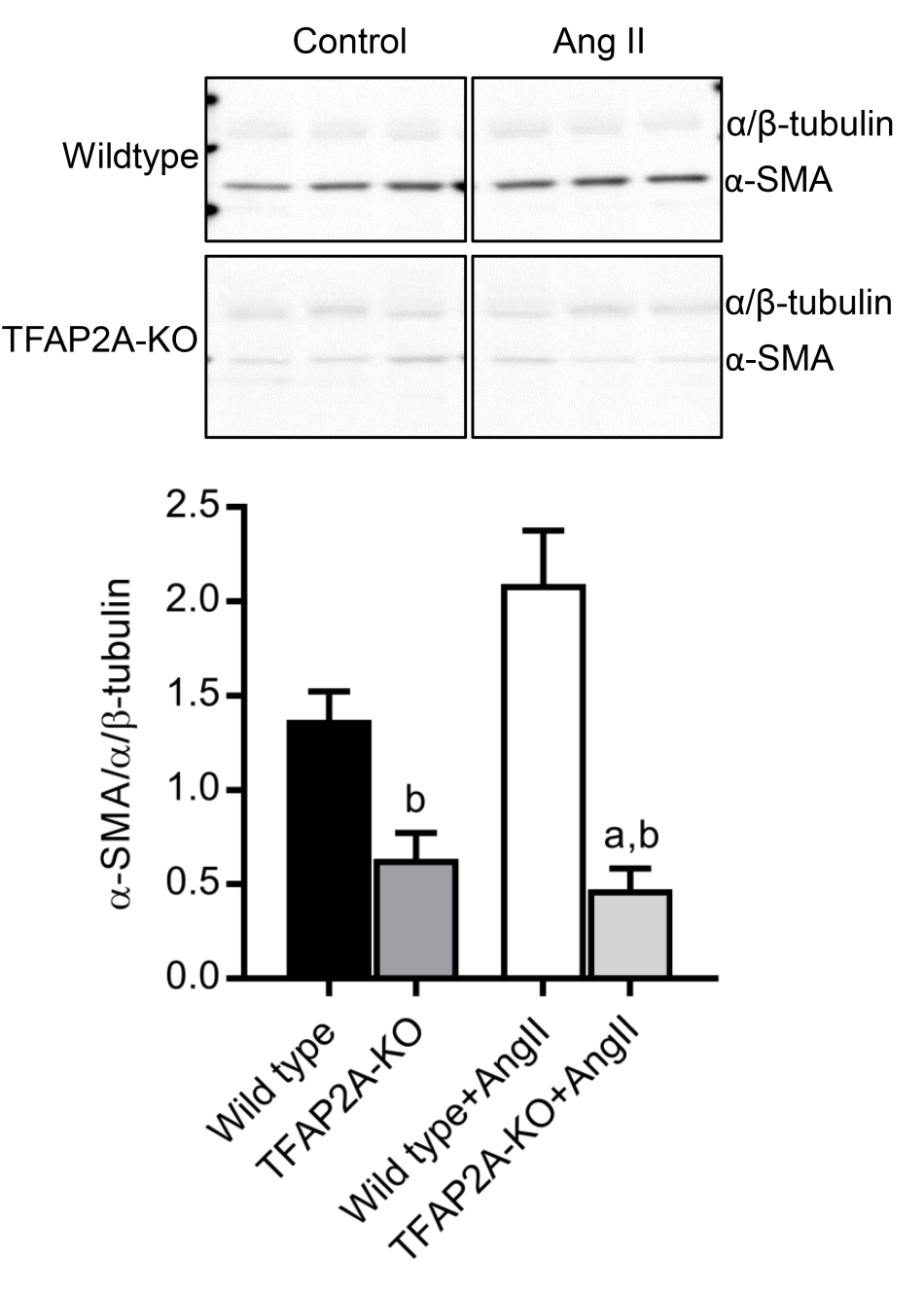

Supplement: Supplementary file 1 [file JCMM-23-6494-s001.docx]
